# Starch-Based Pickering Emulsions as Platforms for Topical Antibiotic Delivery: In Vitro and In Vivo Studies

**DOI:** 10.3390/polym11010108

**Published:** 2019-01-10

**Authors:** Joana Marto, Aida Duarte, Sandra Simões, Lídia Maria Gonçalves, Luís Filipe Gouveia, António José Almeida, Helena Margarida Ribeiro

**Affiliations:** 1 Research Institute for Medicines (iMed.ULisboa), Faculty of Pharmacy, Universidade de Lisboa, 1609-003 Lisbon, Portugal; ssimoes@ff.ulisboa.pt (S.S.); lgoncalves@ff.ulisboa.pt (L.M.G.); lgouveia@campus.ul.pt (L.F.G.); aalmeida@ff.ulisboa.pt (A.J.A.); hribeiro@campus.ul.pt (H.M.R.); 2 Centro de Investigação Interdisciplinar Egas Moniz, Campus Universitário, Quinta da Granja, 2829-511 Monte de Caparica, Portugal; aduarte@ff.ulisboa.pt

**Keywords:** starch, modified starch, Pickering emulsions, minocycline, superficial skin infection, topical application

## Abstract

The present study investigated a new approach to treat superficial skin infections by topical application of minocycline hydrochloride (MH) formulated in a novel starch-based Pickering emulsion (ASt-emulsions). The emulsions were fully characterized in terms of efficacy, as well as in vitro release and permeation studies. The emulsions provided a prolonged MH release, always above its minimum inhibitory concentration against *Staphylococcus aureus*, although the drug did not permeate through the entire skin layer. The in vitro antibacterial activity of MHASt-emulsions against *S. aureus* was confirmed and their therapeutic efficacy was assessed using an in vitro skin-adapted agar diffusion test. In vivo antibacterial activity, evaluated using the tape-stripping infection model in mice, showed the topical administration of MH was effective against superficial infections caused by *S. aureus*. This study supports the potential of ASt-emulsions as promising platforms for topical antibiotic delivery, contributing to a new perspective on the treatment of superficial bacterial infections.

## 1. Introduction

Topical pharmaceutical emulsions are suitable dosage forms to produce topical therapeutic action at specific sites in the skin [1]. Pickering emulsions are suitable drug carriers for topical pharmaceutical applications, because of their non-toxic and non-carcinogenic characteristics and good skin biocompatibility [1,2]. These systems are gaining increasing interest as topical formulations due to: (a) the growing demand for natural and safer products; (b) low cost and environment-friendly processes; (c) the ability to control drug release and promote drug penetration. However, literature on Pickering emulsions reporting in vivo skin permeation and safety studies is rare [2].

The skin is the major organ that protects the body from microorganisms and other environmental pathogens. In injuries such as burns and trauma, the skin becomes vulnerable to microbial invasion due to loss of integrity and local and systemic immunity suppression. As a result, superficial infection may not only delay the wound healing process, but also can cause systemic infection under severe conditions, such as burns and bedsores [3]. The most common Gram-positive bacterial pathogen that colonizes wounds and causes infection is *Staphylococcus aureus*. Although superficial bacterial infections caused by *S. aureus* are usually treated by the oral route, topical antibiotics are used to treat skin infection or as prophylaxis to protect skin from further infection [4].

Minocycline hydrochloride (MH), a long-acting, broad-spectrum tetracycline, is effective in eradicating topical pathogens implicated in superficial infections, especially those caused by Gram-positive bacteria, including multiresistant strains, such as methicillin resistant *S. aureus* (MRSA) [5]. There is a concern regarding MH systemic side effects, mostly derived from long-term treatments by the oral route, namely opportunistic yeast infection, gastrointestinal disturbance and cutaneous symptoms [6]. It has also been reported that drug concentration in skin layers resulting from a single topical application is much higher than those obtained after prolonged oral administration [7,8]. In addition, there is a recent trend for producing novel carriers loaded with antibiotics to increase the drug’s solubility and allow topical application. Literature describes topical dosage forms loaded with MH with concentrations ranging from 0.25–4% [3,9,10]. In fact, numerous MH topical delivery strategies have been proposed using formulations, such as ointments [10], hydrogels [3,5], foams [11] and microspheres [9,12]. Nevertheless, topical MH advanced formulations involving particulate carrier systems still remain in the experimental therapeutic field. In this context, we have proposed starch-based Pickering emulsions as suitable drug carriers for topical pharmaceutical applications.

Thus, the purpose of this study was to develop and fully characterize novel starch-based Pickering emulsions for topical delivery of MH in order to increase its efficacy and safety. The studies included in vitro release and permeation, as well as detailed in vivo efficacy studies, demonstrating the successful application of starch-based Pickering emulsions and highlighting their potential for sustained drug release, acting as a promising vehicle for delivery of topical antibiotics.

## 2. Material and Methods

### 2.1. Materials

Aluminum starch octenylsuccinate (ASt) (DryFlo^®^ Plus) was a gift from AkzoNobel (Amsterdam, The Netherlands). The oils used were liquid paraffin (LP) purchased from Mosselman (Ghlin, Belgium) and caprylic/capric acid triglyceride (Tegosoft^®^ CT) (CT) a kind gift from Evonik Industries AG (Essen, Germany). Minocycline hydrochloride (MH) was obtained from Laboratórios Atral S.A. (Castanheira do Ribatejo, Portugal). Purified water was obtained by reverse osmosis and electrodeionization (Elix 3, Millipore, MA, USA) being afterwards filtered (pore 0.22 µm).

### 2.2. Methods 

#### 2.2.1. Preparation of Minocycline Hydrochloride (MH)-Loaded Starch-Stabilized Pickering Emulsions (MHASt-Emulsions)

Six w/o emulsions stabilized by ASt granules were prepared according to the development studies described in a previous publication [1]. Briefly, the ASt particles were first dispersed in the oil phase, using a vortex mixer until total dispersion. The oil and aqueous phases were then mixed together with an UltraTurrax^®^ T25 homogenizer (IKA^®^-Werke GmbH & Co. KG, Staufen, Germany). The qualitative and quantitative compositions of the MHASt-emulsions are described in Table 1.

The microscopic aspect of MHAst-emulsions was confirmed. The droplet size was determined using the image analysis software Olympus Stream Essentials^®^ (Tokyo, Japan). All formulations were stored during 12 weeks at 25 ± 2 °C, protected from the light by aluminium foil. Samples were analyzed for macroscopic appearance before the storage period and on 1, 2, 3, 4 and 12 weeks of storage. Macroscopic appearance was assessed by visual inspection. 

#### 2.2.2. In Vitro Release Studies

Release studies of MH from Pickering were performed in finite dose conditions using hydrophilic polysulfone membranes filters (Tuffryn^®^, 0.45 µm; Pall Corporation, New York, NY, USA), using Franz diffusion cells with 1 cm^2^ surface area. The receptor phase consisted of purified water. The system was maintained at 37 ± 2 °C and samples (0.3 ± 0.1 g) were applied evenly on the surface of the membrane in the donor compartment and immediately sealed with Parafilm^®^ to prevent evaporation. Samples of the receptor phase (200 µL) were collected at predefined times (1, 2, 4, 8, 12 and 24 h). Release tests were performed using 6 Franz cells per formulation. The samples were analyzed using HP 1100 series System liquid chromatography (VWR, Radnor, PA, USA) equipped with a pump (G1310A), an autosampler (G1329A) and an UV detector (G1328A). The auto sampler chamber was maintained at room temperature. Injection volume was 10 µL and purified water at a flow rate of 1.0 mL/min was used as sample carrier. The running time was 0.5 min and detection was made at 280 nm. Peak areas were estimated using the Value Solution ChemStation software (VWR, Radnor, PA, USA).

The data obtained from in vitro release studies were computed using DDsolver [13], which is an Excel-plugin module, and the resultant data were fitted to five different kinetic models [14]: zero order, first order, Higuchi model, Korsmeyer–Peppas and Weibull. In all models, F is the fraction (%) of released drug in time, *t*. The adjusted coefficient of determination (R^2^_adjusted_) was estimated for each model, fitted and used as a model ability to describe a given dataset. 

The dissolution efficiency (DE) was also calculated from the area under the dissolution curve up to a certain time *t* [15]. 

#### 2.2.3. Topical Delivery Studies of MH

##### In Vitro Skin Permeation and Retention

The skin permeation of MH was measured using Franz diffusion cells and newborn pig skin obtained from a local slaughterhouse. The entire skin was cut into sections (1 cm^2^ permeation area). Purified water was used as the receptor phase that assured perfect sink conditions during the whole experimental period. According to solubility studies, purified water allowed maximal solubility for MH (618 μg/mL). The cells were immersed in a bath system at 37 ± 2 °C under stirring (200 rpm). The formulations samples were applied on the skin surface in the donor compartment. Samples were collected from the receptor fluid at pre-determined time points and replaced with an equivalent amount (200 µL) of fresh receptor medium. The MH content in the withdrawn samples was determined by flow injection. 

The in vitro skin retention study was performed by tape stripping according to the method recommended by OECD Guideline 428 [16]. After 24 h, the stratum corneum (SC) was removed using 20 adhesive tapes (Scotch^®^ 3M, Bracknell, UK). All the tapes with the removed SC and the remaining skin (viable epidermis and dermis—ED) were cut into small pieces used for the extraction process previously validated. The final solution was centrifuged (30,000 rpm, 10 min) and the supernatant was filtered and assayed as above described to quantify the amount (%) of MH retained in these skin layers (SC + ED).

#### 2.2.4. In Vitro Determination of the Antibacterial Activity of MH in ASt-Emulsions

The standard curve and the minimum inhibitory concentration (MIC) of MH against *Staphylococcus aureus* ATCC 6538 was first determined. Different concentrations of MH solutions were prepared in sterile distilled water and tested against *S. aureus* using the standard disc diffusion method (DDM), in accordance with the Clinical and Laboratory Standards Institute Standards (CLSI) guidelines [17]. 

To accomplish this, the microorganism inoculum was prepared from 18 h broth culture and the suspension was then adjusted to a turbidity of 0.5 McFarland (~1.5 × 10^8^ colony-forming units (cfu/mL). Then, the Mueller-Hinton agar (MHA) (Thermo Scientific™ Oxoid™, Hampshire, UK) was poured into petri dishes and inoculated with 100 μL of the suspension containing ~1.5 × 10^8^ cfu/mL of bacteria. Sterile paper discs (6 mm; Thermo Scientific™ Oxoid™, Hampshire, UK) were loaded with 15 μL of MH solution with different concentrations, ranging from 17 to 545 µg/mL. After incubation at 37 °C for 24 h, the mean of the inhibition zone diameters and the standard deviation were calculated and, the lowest concentration of MH capable of inhibiting growth after 24 h of incubation was then recorded as the MIC. 

The antibacterial activity of the MH on the different ASt-emulsions was tested using the DDM described by the CLSI. MH was used as the positive control and a blank disc was used as the negative control. 

#### 2.2.5. Skin Adapted Agar Diffusion Test

This method is an adaptation of a well-known in vitro assay (DDM) to verify the effectiveness of antibiotic product contained in topical formulations and is described in detailed elsewhere [18]. An aliquot of 15 μL of test formulations were applied on skin discs that were then incubated on a mannitol salt agar plate (MSA) (Thermo Scientific™ Oxoid™, UK) inoculated with *S. aureus* 24 h at 37 °C. After incubation time, the efficacy of each tested formulation was determined by measuring the inhibition zone diameter.

#### 2.2.6. In Vitro Cytotoxicity Studies

##### Cell Culture Conditions and Cytotoxicity Assays

To determine in vitro emulsions effects on cell viability, HaCat cells were incubated with ASt-emulsions and MHASt-emulsions for 24 h and cell viability was determined using the MTT assay as described in detail elsewhere [1]. The percentage of viable cells was established relative to cells treated with the vehicle, for the drug in solution, and for the drug in emulsions. Inhibitory concentrations (IC50) were calculated using GraphPad Prism^®^ software v5.0 (GraphPad Software, Inc., San Diego, CA, USA) by the sigmoidal curve fitting method.

#### 2.2.7. Scratch Wound Healing Migration Assay

Cells were seeded onto six-well plates and cultured until high confluence. The monolayer was wounded by scraping it with a sterile tip. The wells were washed twice with medium without serum and then finally with complete culture medium. The healing process was examined during this time with an optical microscope and the wounds microphotographs were obtained at times of 0, 24 and 48 h after making the wound. The area of the wounds for the different time-points were compared with the wound area at time 0, in order to evaluate the migration rate of cells.

#### 2.2.8. In Vivo Studies

All animal experiments were carried out with the permission of the local animal ethical committee in accordance with the European Union (EU) Directive (2010/63/EU), Portuguese law (DL 113/2013) and all relevant legislations.

##### 2.2.8.1. In Vivo Antibacterial Activity Studies: Tape-Stripping and Infection Model

Animal tape-stripping and infection experiments were performed according to Kugelberg et al. [19], using eight-week-old female BALB/c mice (Instituto Gulbenkian de Ciência, Oeiras, Portugal). Mice were anesthetized by intraperitoneal injection of a mixture of ketamine (75 mg/kg body weight) and medetomidine (1 mg/kg body weight), the hair of the dorsum, midline, was clipped and the skin was stripped 4 times with adhesive tapes (Transpore™, 3M) of ca. 4 cm^2^. After stripping, a bacterial infection was initiated by placing on the skin 20 µL containing ~3.7 × 10^8^ cfu/mL of *S. aureus* ATCC 6538 culture from an overnight culture. Six groups of animals, each containing 5 mice were used. The infected mice were treated with: 10 mg/mL clyndamicine phosphate topical solution (Commercial solution (CS)—Dalacin^®^ T, Pfizer, New York, NY, USA) (Group 1); ASt-emulsion with LP (Group 2); MHASt-emulsion with LP (Group 3); ASt-emulsion with CT (Group 4); MHASt-emulsion with CT (Group 5); and a group of infected mice remained untreated working as the infection control (Group 6). An additional group of three stripped mice but not infected was used to control the stripping damage on the skin. Typical colonies were then determined as the total number of cfu/mL using log10-transformed data. Data were analyzed using the GraphPad PRISM^®^ 5 software (San Diego, CA, USA) and the groups were considered significant when the estimated p values were lower than 0.1 (the chosen α error), to increase statistical power.

##### 2.2.8.2. Skin Histology

The histological features of the lesions were assessed in the same groups of mice described in Section 2.2.8.1. For this purpose, at the end of those experiments, animals were sacrificed with CO_2_ narcosis, skin wounds were excised with a wide margin of normal skin (approx. 2 mm) and processed for routine histology. After fixation, trimming was performed longitudinally, in the direction of the hair flow and centered on the wound. Samples were embedded in paraffin, sectioned at 4 µm, and stained with hematoxylin and eosin (H&E). Histopathological analysis was performed by a pathologist blinded to experimental groups using a Nanozoomer SQ slide scanner and NDP.view2 software (Hamamatsu Photonics, Hamamatsu, Japan). Standard criteria were used to stage the lesion and to perform semi-quantitative analysis of the inflammatory cell infiltration [20].

#### 2.2.9. Statistical Analysis

Two or one-way analysis of variance (ANOVA) when appropriate and the Tukey–Kramer post-hoc multiple comparison test were used to identify the significant differences between the groups and were performed using GraphPad PRISM^®^ 5 software. An α error of 5% or 10% (for in vivo studies) were chosen to set the significance level unless stated otherwise.

## 3. Results and Discussion

### 3.1. Formulation Development 

In a previous work we described the optimization of experimental conditions to obtain stable and pharmaceutically acceptable w/o ASt-emulsions suitable for hydrophilic drugs [1]. That work clearly indicated that ASt-emulsions were highly influenced by the amount of starch and the lipid type, with self-preserving properties. MH is a hydrophilic tetracycline, with a molecular weight of 493.9 Da and a LogP of −0.65. However, it presents increased lipophilicity when compared to the other tetracyclines, which enhances minocycline penetration into various tissues [21]. It was chosen for its broad spectrum activity against many aerobic and anaerobic Gram-positive and Gram-negative bacteria. In addition it has a wide range of potential clinical indications such as respiratory tract infections, sexually transmitted infections, acne vulgaris and other skin infections [21], showing also other meaningful pharmacological effects, including anti-inflammatory action [5]. 

Optical microscopy images of MHASt-emulsions revealed the emulsions’ microstructure and showed that MH does not influence the droplet size distribution when compared with ASt-emulsions [1]. Moreover, in 7.5%-MHASt-emulsions, several small inner drops of water, with homogeneous size were observed being dispersed in the oil phase. The MHASt-emulsions with low and intermediate amounts of ASt, exhibited a heterogeneous structure with larger inner oil droplets. The droplet size differences between these emulsions are probably due to insufficient coverage at the oil–water interface. Thus, aggregation of droplets may lead to coalescence and the formation of larger droplets until the phases become separated. 

The macroscopic characteristics of MHASt-emulsions were assessed by visual inspection throughout 12 weeks at 25 ± 2 °C. It was concluded that the MH does not influence the MHASt-emulsions stability, since no more coalescence or phase separation were observed in these emulsions when compared with ASt-emulsions. In the present study, coalescence occurred firstly in the emulsions with the lowest level of ASt, in the second week of storage, followed by phase separation on week 4. This also occurred with the emulsions prepared with the central level of ASt. The 7.5%-MHASt-emulsions, with the highest level of ASt, maintained their stability during 12 weeks after preparation. With sufficient coverage at the interface, which is the case of α-MHASt-emulsions, ASt particles act as barriers against droplet coalescence that enhance the emulsion stability [22,23]. Unlike surfactants, ASt particles do not reduce the oil-water interfacial tension, but they strongly get adsorbed at the oil-water interface. However, particle adsorption on the oil–water interface is a slow process and needs to be enhanced by mixing [24]. 

### 3.2. In Vitro Release Studies 

**Figure 1:**
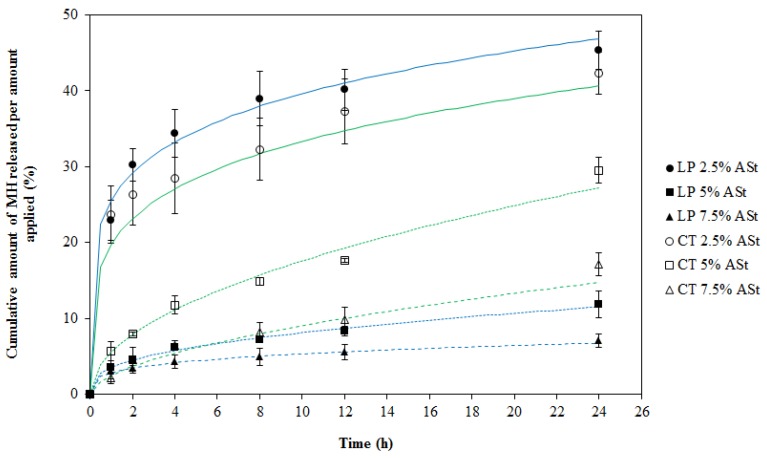
Release profiles and fitting curves of Korsmeyer–Peppas model for MH from MHASt-emulsions through Tuffryn^®^ membrane in water at 37 °C (mean ± SD, n = 6). LP—MHASt-emulsions with LP; CT—MHASt-emulsions with CT.

The MH release profiles from the Pickering emulsions, as a function of time, during 24 h, through the Tuffryn^®^ membrane are shown in Figure 1. Drug release was significantly depended on the amount of starch and also on the type of lipid type (*p* < 0.05). 

In order to achieve a controlled MH release, it is crucial to have the drug strictly confined in the water droplets of w/o MHASt-emulsions. Therefore, the choice of LP and CT as continuous phase was motivated by the solubility of MH in both external phases that makes the partition coefficient of MH between aqueous and oil phases very low. As mentioned above, its low LogP value contributes to keep MH restricted to the inner water phase of the emulsion, which is apparently confirmed by the absence of any burst effect at the initial moments of the release study.

After 1 h, the MH release was higher from emulsions containing lower amounts of starch and, at the end of the study (24 h), when comparing emulsions with the same starch concentration. Moreover, the emulsions with lower starch concentrations (2.5%) showed a faster release at the early stages of the study, which may be due to the incomplete coverage by ASt particles at the interface that allowed an easier diffusion of MH from the droplet. In contrast, MH release from the emulsions with high starch concentrations, especially 7.5%, was considerably slower, suggesting that the shell of starch particles around the droplets efficiently acted as a barrier to interfacial diffusion. This barrier might delay the release by the presence of “starch-packed” granules and the increased viscosity. On the other hand, the extent of drug release was often greater from formulations comprising low starch concentrations.

Therefore, it is possible that the mere encapsulation of MH inside the droplets of ASt-emulsions will result in a slow release profile controlled by interfacial diffusion. The MH transport mechanism may also be affected by drug adsorption at starch surface as described elsewhere [25]. In this context, the kinetics and mechanisms of MH release were studied using the first order, Higuchi, Korsmeyer–Peppas and Weibull kinetic models. The results showed that the Korsmeyer-Peppas kinetic model best fitted the 24 h release profile as confirmed by the highest values for R^2^_adjusted_ and model selection criterion (MSC) and lowest values for the Akaike information criterion (AIC) [13]. The determined values of diffusion exponent (n) ranged between 0.18 and 0.49 (Table 2), indicating the drug release from these MHASt-emulsions followed a Fickian type of diffusion, indicating the rate-controlling mechanism in the drug release process is the diffusion of the dissolved drug through the vehicle network to the external medium, which is in agreement with the literature [26,27]. Therefore, at this stage, release data do not support a significant contribution of the drug adsorption at the starch surface.

In the case of 2.5%-ASt-emulsions, the low starch concentration led to the formation of a looser interfacial barrier that facilitated MH release and increased DE_24 h_ (38.4 ± 5.4% and 40.1 ± 8.3%, for LP and CT, respectively). Comparing MHASt emulsions with different lipid type, a higher dissolution efficiency was found for the emulsions with CT when compared to those containing LP, because the LP (hydrocarbon family) is more lipophilic than CT (mixed triester of glycerin and caprylic and capric acid) (Table 2).

Overall, these results justify the existence of a relation between starch concentration, lipid type and MH release, confirming those factors as the major parameters affecting ASt-emulsions, which corroborate our previous report [1].

### 3.3. Topical Delivery Studies

No retention was detected in the newborn pig skin within 24 h of exposure and MH did not permeate the entire skin layer, suggesting a minimal potential for the systemic absorption of MH upon topical administration. However, it is important to understand how Pickering emulsions, as practical multifunctional vehicles, can be used to modulate drug penetration through the SC and control drug release at different skin sites [22]. 

Several studies demonstrated the possibility of the emulsion stabilizing particles to increase the drug penetration over the skin [22,25]. Factors affecting transdermal transport from emulsions are not yet fully understood. Formulation can alter simultaneously the properties of the skin and the activity of the substance. In previous studies, Pickering emulsions have increased the penetration of hydrophilic drugs when compared to traditional formulations [27]. Authors have postulated a better adhesion of the droplets to the skin surface and penetration of the particles into the skin [27]. Particles smaller than 3 µm penetrate both by diffusion through the SC and by the hair follicles [28]. On the other hand, particles ranging from 3 to 10 µm mainly penetrate into hair follicles, while particles larger than 10 µm remain on the skin surface, which is the case of ASt particles herein studied [29,30]. Thus, it is not expected that ASt particles may act as skin carriers in MHASt-emulsions.

Hydration of the SC is also a major mechanism to increase the penetration of most active molecules, as water has the ability to open up the compact structure of the horny layer. The water content of the horny layer can be increased either by delivering water from the vehicle to the skin or by preventing water loss from the skin when partially occlusive formulations are applied to the skin, becoming an important skin permeation pathway for hydrophilic molecules. 

Thus, the long-term topical application of w/o emulsions increases the water retention and increases skin hydration, providing a healthier skin barrier with a higher permeation aptitude [31]. Concerning the external phase, polar lipids, such as CT, greatly increase penetration into the skin [32]. On the other hand, LP is a non-polar solvent, creating an occlusive barrier onto the membrane. It was demonstrated that the occlusion effect is widely used to enhance the penetration of applied drugs in clinical practice [33]. However, occlusion does not increase percutaneous absorption of all chemicals, being more effective for lipid-soluble, non-polar molecules but less efficient for polar molecules, which is the case of MH [34]. 

Regarding highly lipophilic molecules, Pickering emulsions promote high accumulation in the SC. The practical utility of such behaviour is either preventing skin penetration of molecules that should be retained at the skin surface, e.g., sunscreens and topical antibiotics, or using the SC as a reservoir for slow release of the drug to the deeper skin layers [35,36]. Therefore, regarding a pharmaceutical application, w/o Pickering emulsions are particularly indicated for sustained and targeted drug release to the viable epidermis, forming a drug reservoir in the deeper layers of SC. Such mechanism has been claimed for nano- and microparticles intended for skin drug delivery [37].

### 3.4. In Vitro Determination of the Antibacterial Activity of MH in ASt-Emulsions

In order to test the potential of developed formulations as an antimicrobial drug candidates it is important to establish their effectiveness in vitro. The in vitro antibacterial activity of MH in MHASt-emulsions was tested against *S. aureus* ATCC 6538. All tested MHASt-emulsions produced a mean inhibition zone after 24 h incubation between 14.9–22.8 mm, which exceeded that of the calculated MIC value for MH (13.4 mm) against the same microorganism (Table 3). So, the amount of MH released drug from all emulsions exceeded the MIC towards the tested microorganism (0.190 µg/mL). The blank ASt-emulsions showed no growth inhibition of microorganism. These results corroborate with the results from the in vitro release studies, confirming the low starch concentration led to the formation of a looser interfacial barrier that facilitated MH release. 

### 3.5. Skin Adapted Agar Diffusion Test

**Figure 2:**
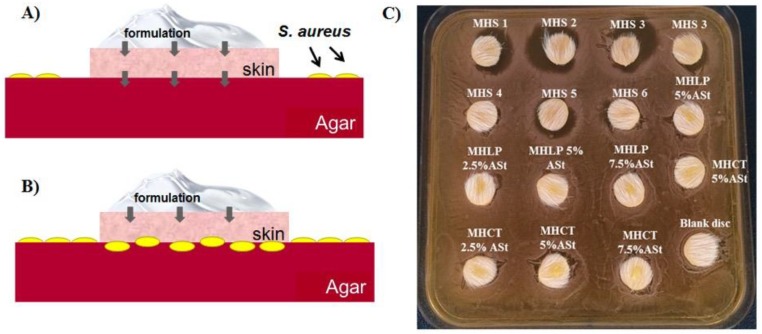
Skin adapted agar diffusion test cross sections. (**A**)—Drug skin permeation (black arrows) inhibits *S. aureus* growth and an inhibition zone is observed around the skin disk. (**B**)—Drug skin retention (black arrows) does not inhibit *S. aureus* growth. No inhibition zone is observed under the skin nor around the skin disk. (**C**)—Petri dish for the skin adapted agar diffusion test. MHS1 to MHS6—Solution of MH ranging from 545 to 17 µg/mL. MHLP—MHASt-emulsion with LP; MHCT—MHASt-emulsions with CT.

The skin adapted agar diffusion test intends to assess local susceptibility rates, the diameter of the inhibition zone being proportional to the sensitivity of the microorganism and the efficacy of the antibacterial drug [38]. Figure 2A,B illustrate the procedure and the two possible outcomes of this test. In fact, the test revealed no drug skin permeation occurred when MHASt-emulsions were applied onto the skin disks (Figure 2C), i.e., the situation illustrated in Figure 2B, which corroborate the MH permeation studies measurements, with no zones of inhibition observed around or underneath the skin disks. However, aqueous MH solutions promoted zones of inhibition, between 2 and 10 mm, surrounding the skin discs, clearly showing the diffusion of MH and lower skin adhesion. Since MH was confined inside the water droplets of MHASt-emulsions, direct transfer to the skin may take place if water droplets are deposited on the skin surface, confirming earlier findings reported by other authors [25], who demonstrated that w/o Pickering emulsion droplets adhered better to the skin surface than the water droplets. 

Based on these results, and on the data obtained from stability and in vitro release studies, the 5%-ASt-emulsions and 5%-MHASt-emulsions were selected for subsequent in vivo studies. 

### 3.6. In Vitro Cytotoxicity Studies 

To predict the potential cytotoxicity of the MHASt-emulsions, cell viability was evaluated using HaCaT cell line in a MTT assay. Firstly, the IC50 of the drug in solution was determined by a non-linear regression analysis, and subsequently, the found concentration was used to determine the cell viability after application of the emulsions. The cytotoxicity of MH dissolved in water (525 μg/mL) was also analyzed.

**Figure 3:**
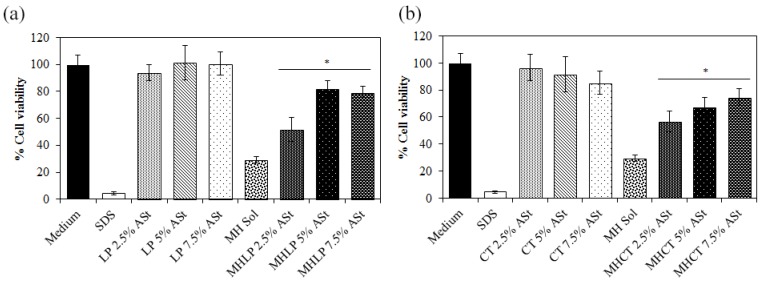
Viability of HaCaT cells after 24 h of incubation with MH at the concentration of 525 μg/mL, either in the free form or incorporated into the emulsions (**a**) LP (**b**) CT; SDS—sodium dodecyl sulfate (mean ± SD, n = 10) (* *p* < 0.05).

Cell toxicity was different according to the way MH was presented to the HaCat cells, i.e., dissolved in water or formulated in ASt-emulsions. The IC50 of MH in aqueous solution was 50.9 ± 1.0 µg/mL, while the same concentration in the form of a Pickering emulsion decreased the cytotoxicity (Figure 3). Therefore, the incorporation of MH in ASt-emulsions protects human keratinocytes from the cytotoxicity of the drug. Cell viability increased to more than 50%, depending on the starch concentration. As we can see in Figure 3, drug diffusion to the culture medium started to happen in the emulsions with 2.5% ASt, decreasing cell viability, and this could be due to the fact that with less starch, the interface between oil and water might not be fully covered, leading to an earlier drug diffusion. 

According to the Organisation for Economic Co-operation and Development (OECD) an irritant substance is predicted if the mean relative tissue viability is found below 50% of the mean viability of the negative controls for a 15–60 min of exposition time [39]. Thus, the MHASt-emulsions prepared with both lipid components can be intended as non-irritant and the amounts of starch and MH used can be considered as safe. These results are in line with findings described in the literature, whereby starch concentrations as high as 30.5% are devoid of skin irritation or sensitization properties [40]. 

Moreover, emulsions stabilized by solid particles can be distinguished from emulsions stabilized with classic emulsifiers concerning their irritative potential for the reason that emulsifiers have the potential to act as penetration enhancers by disturbing SC integrity and hence may enable or enhance diffusion of other molecules through the skin [41,42]. 

### 3.7. In Vitro Scratch Wound-Healing Migration Assay

**Figure 4:**
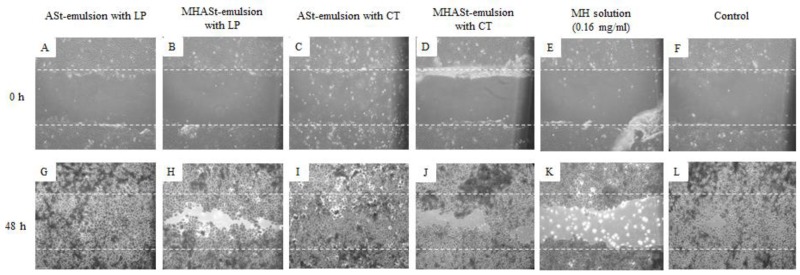
Representative time-lapse images of HaCaT keratinocyte scratch assays immediately after the scratches had been made and then after 48 h in the presence of 5%-emulsions with LP (**A**,**G**), 5%-MHASt-emulsions with LP (**B**,**H**), 5%-ASt-emulsions with CT (**C**,**I**), 5%-MHASt-emulsions with CT (**D**,**J**), MH solution (**E**,**K**) or control medium (**F**,**L**). The cells were allowed to migrate for 48 h, fixed and photographed. Outlines of the original wounds are marked with dashed lines. Original magnification 40×.

In vitro scratch wound healing assays were also performed for MHASt-emulsions using HaCaT cells in the presence or absence of MH. In order to evaluate the influence of ASt-emulsions and MH on the wound healing, 5%-ASt-emulsions were studied. In untreated HaCaT cells, MHASt-emulsions and MH solution did not repair scratch wounds after 48 h, but treatment with 5%-ASt-emulsions led to complete closure of the wound in 48 h (Figure 4). Thus, irrespective of the addition of MH, ASt-emulsions greatly improved wound healing. In addition, there was no significant difference between the ASt-emulsions with LP and CT or between MHASt-emulsions with LP and CT. According to several authors, modified starches have been reported to accelerate wound healing and presented highly absorptive capacity [43,44]. On the other hand, the migration of HaCaT cells could be blocked by MH due to its cytotoxicity (Figure 4). 

### 3.8. In Vivo Studies

#### 3.8.1. In Vivo Antibacterial Activity Studies: Tape-Stripping Infection Model

**Figure 5:**
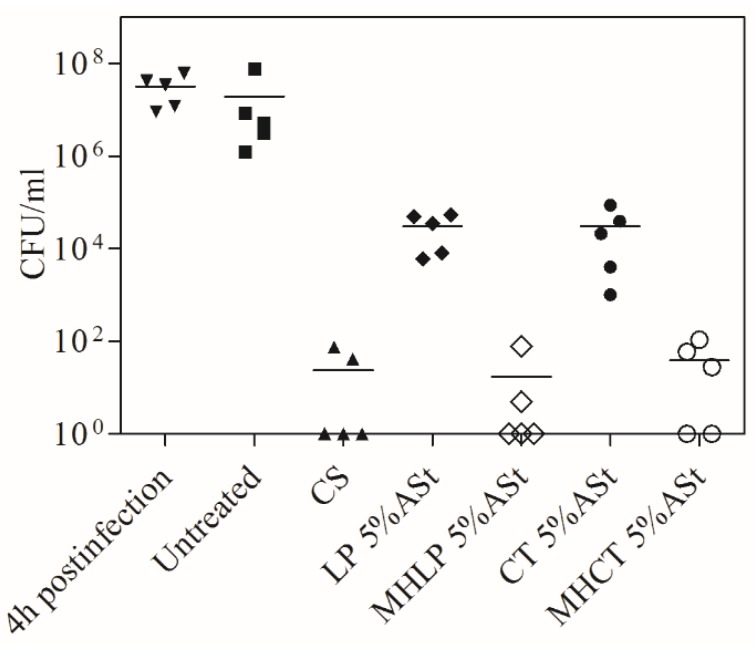
Effect of different formulations on antibacterial activity. Tape-stripped mice were infected with *S. aureus* ATCC 6538. The number of bacteria (cfu/mL) extracted from each mouse is represented by the symbol for the corresponding experimental group. The median value of the data for each group is shown as a horizontal bar. CS: Commercial solution.

The number of cfu recovered from the skin 4 h post-infection, i.e., after application of ~3.7 × 10^8^ cfu/mL of *S. aureus* ATCC 6538 is shown in Figure 5. The different treatment regimens started after this initial 4 h period. There was no significant difference (*p* < 0.1) in the numbers of cfu/mL when 4 h versus 4 days of untreated mice were compared (6.83 ± 0.67 log10), demonstrating the successful establishment of a staphylococcal infection in this model (Figure 5). The comparison of the treatments with ASt-emulsions and MHASt-emulsions revealed a reduction in the numbers of cfu/mL that was statistically significant (*p* < 0.1). Therefore, 5%-MHASt-emulsions provided in vivo MH concentrations that exceed the MIC of susceptible *S. aureus*. In this study, it was observed that 4 days of topical treatment with MHASt-emulsions resulted in significantly reduced bacterial loads (Figure 5). 

The present study demonstrated that MHASt-emulsions were highly efficient in eradicating *S. aureus*-induced superficial infection, suggesting the possibility of replacing systemic antibiotic treatment by local treatment. Furthermore, topical treatment may have an advantage over systemic treatment to achieve rapid reduction of *S. aureus* bacterial loads in superficial skin infections. This would result in decreased drug exposure and associated side effects, potentially increasing patient compliance. In addition, a rapid bacterial death and short therapy regimens with MHASt-emulsions could ultimately result in reducing treatment costs and minimizing bacterial resistance [19]. 

#### 3.8.2. Skin Histology

**Figure 6:**
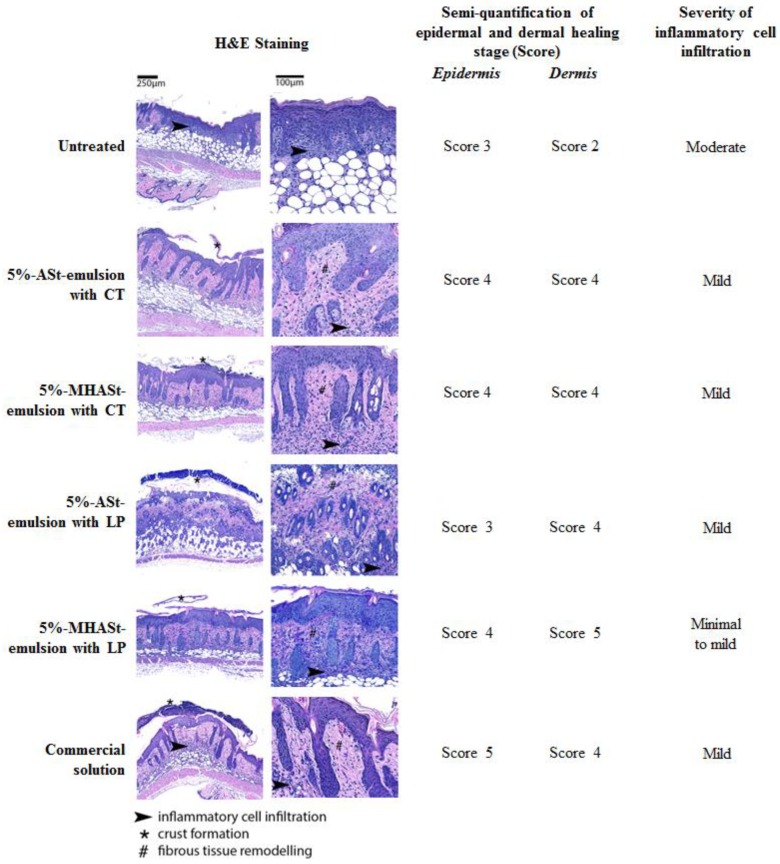
Hematoxylin and eosin-stained sections of tape-stripping lesions in BALB/c mice. Experimental groups included: untreated; 5%-ASt-emulsion with CT; 5%-MHASt-emulsion with CT; 5%-ASt-emulsion with LP; 5%-MHASt-emulsion with LP; Commercial solution; all previously preceded by epicutaneous bacterial infection.

Histological analysis of wounds included staging the wound-healing process and scoring the extent/severity of inflammatory cell infiltration, as shown in Figure 6. 

Epidermal reparation is a crucial step in cutaneous restoration of irritated skin and wound healing. It involves the combination of two cellular processes, i.e., proliferation and migration of epidermal cells. Simultaneously, dermal reparation occurs and generally follows several stages: inflammation, tissue formation, and finally remodelling [20]. In the present study, re-epithelization of the epidermis was seen in all groups. Untreated mice displayed severe hyperplasia, with crust formation, still with no hair follicle or adnexal gland differentiation/formation, and with important inflammatory cell infiltration and active tissue formation in the dermis. In contrast, for all other groups, epidermal healing was generally at advanced stages of healing, hyperplastic but already with hair follicles and adnexal gland formation; the dermis was at late stages of remodelling and inflammatory cell infiltration was generally mild. In comparison with all other treatment groups, MHLP0 mice showed mildly decreased inflammatory cell infiltration. It was demonstrated that MH could markedly reduce the in vivo inflammatory activity [45].Thus, the present study corroborates the concomitant anti-inflammatory synergistic action on MH antibacterial activity. 

In the groups treated with the starch-based formulations (groups 5%-ASt-emulsion with CT and 5%-MHASt-emulsion with CT), the epidermis was seen to display prominent irregular hyperplasia of the epithelium with tongue-like epithelial projections into the dermis (Figure 6). This is a reactive pattern of hyperplasia, which contrasts with the epidermal hyperplasia typically seen in the setting of wound healing and in all other groups, and appears to be induced by this vehicle. Only scarce bacteria were seen at the wound surface, in all groups. Therefore, MH-containing w/o Pickering emulsions may be a useful and promising approach for the treatment of superficial bacterial infection such as rosacea and impetigo [46,47].

## 4. Conclusions

The present study demonstrated that ASt-emulsions are suitable systems for topical MH formulation, presenting simple composition and allowing drug solubilization and deposition onto the skin, with a prolonged drug release above the MIC of MH against *S. aureus*. The MH did not permeate through the entire skin layer, suggesting a minimal effect for the systemic absorption of the MH in topical administration. Furthermore, MHASt-emulsions have no toxicity on HaCat cells in the concentration range of its anti-staphylococcal MIC. The topical administration of MH in ASt-emulsions provides drug-delivery directly to the infected lesion site, and leads to fast treatment, while avoiding the side effects common in oral MH treatment. These properties make MHASt-emulsions a suitable carrier to be used against superficial *S. aureus* skin infections.

In conclusion, starch-based Pickering emulsion technology seems to be a potential candidate for further research studies and, in the future, for expanding the pharmaceutical market of topical antibiotics.

## Figures and Tables

**Table 1:** Qualitative and quantitative composition of the optimized minocycline hydrochloride (MH)-loaded starch-stabilized pickering emulsions (MHASt-emulsions).

Formulations	Quantitative Composition (%, *w*/*w*)											
	ASt-Emulsion with Liquid Paraffin (LP)			ASt-Emulsion with Caprylic/Capric Acid Triglyceride (CT)			MHASt-Emulsion with LP			MHASt-Emulsion with CT		
	2.5%	5%	7.5%	2.5%	5%	7.5%	2.5%	5%	7.5%	2.5%	5%	7.5%
Phase A (external)												
Liquid paraffin	72.50	70.00	67.50	-	-	-	72.45	69.95	67.45	-	-	-
Caprylic/capric acid triglyceride	-	-	-	72.50	70.00	67.50	-	-	-	72.45	69.95	67.45
Phase B (solid particles)												
Aluminum starch octenylsuccinate	2.50	5.00	7.50	2.50	5.00	7.50	2.50	5.00	7.50	2.50	5.00	7.50
Phase C (internal)												
Purified water	25.00	25.00	25.00	25.00	25.00	25.00	25.00	25.00	25.00	25.00	25.00	25.00
Minocycline hydrochloride	-	-	-	-	-	-	0.05	0.05	0.05	0.05	0.05	0.05

**Table 2:** Kinetic parameters obtained after fitting the release data from the MHASt-emulsions with LP and CT to different release models (mean ± standard deviation (SD), n = 6).

	Model	K	R^2^_adjusted_	AIC	T50% (min)	T90% (min)	DE_24 h_ (% ± SD)
2.5%-MHASt-emulsion with LP	First order	0.05	0.723	55.3	14.3	47.5	38.4 ± 5.4
	Higuchi	14.68	0.731	38.3	12.0	39.0	
	Korsmeyer–Peppas	25.63	0.973	25.6	56.3	2388.9	
		n—0.18					
	Weibull	α—3.14 β—0.20 Ti—0.53	0.972	26.3	83.9	12432.0	
5%-MHASt-emulsion with LP	First order	0.01	0.838	30.4	111.1	369.1	8.1 ± 0.2
	Higuchi	2.53	0.960	16.6	390.1	1263.9	
	Korsmeyer–Peppas	3.53	0.989	4.2	13,683.2	185,438.2	
		n—0.39					
	Weibull	α—27.17	0.985	5.1	148,476.5	1,028,8670.6	
		β—0.35					
		Ti—0.40					
7.5%-MHASt-emulsion with LP	First order	0.00	0.273	24.5	182.2	605.1	5.3 ± 0.9
	Higuchi	1.63	0.871	13.1	979.1	3172.1	
	Korsmeyer–Peppas	2.84	0.997	9.3	71,257.0	723,399.2	
		n—0.27					
	Weibull	α—31.79	0.996	11.7	902,911.8	39,677,378.5	
		β—0.24					
		Ti—0.40					
2.5%-MHASt-emulsion with CT	First order	0.05	0.139	50.1	15.8	52.6	40.1 ± 8.3
	Higuchi	11.99	0.775	40.7	18.2	58.8	
	Korsmeyer–Peppas	25.10	0.988	22.2	38.8	743.7	
		n—0.20					
	Weibull	α—3.31	0.974	28.3	283.7	2577.2	
		β—0.21					
		Ti—0.40					
5%-MHASt-emulsion with CT	First order	0.02	0.886	31.6	43.8	145.7	17.9 ± 0.3
	Higuchi	5.65	0.981	18.3	78.4	254.0	
	Korsmeyer–Peppas	5.60	0.988	14.4	85.8	291.9	
		n—0.40					
	Weibull	α—14.72	0.974	22.1	153.9	1015.0	
		β—0.47					
		Ti—0.42					
7.5%-MHASt-emulsion with CT	First order	0.01	0.8693	25.6	94.5	313.8	9.3 ± 1.7
	Higuchi	2.91	0.9884	8.6	317.0	1027.2	
	Korsmeyer–Peppas	2.50	0.9967	0.5	228.4	660.9	
		n—0.49					
	Weibull	α—33.44	0.9846	11.8	507.4	2717.3	
		β—0.51					
		Ti—0.40					

K—release constant; R^2^_ajusted_—adjusted coefficient of determination; AIC—Akaike Information Criterion; T50%—time required for 50% dissolution; T90%—time required for 90% dissolution; DE_24 h_—dissolution efficiency at 24 h of release study; n—release exponent; α—scale parameter; β—shape parameter; Ti—location parameter.

**Table 3:** Comparison of zone of inhibition produced by MHASt-emulsions after 24 h of incubation (mean ± SD, n = 3).

Formulations		Zone of Inhibition (mm)	Concentration of MH Released (µg/mL)
MHASt-emulsions with LP	2.5% ASt	22.8 ± 0.7	1.90 ± 0.12
	5% ASt	19.3 ± 0.2	1.12 ± 0.03
	7.5% ASt	14.9 ± 0.3	0.45 ± 0.05
MHASt-emulsions with CT	2.5% ASt	22.5 ± 0.4	1.88 ± 0.04
	5% ASt	20.1 ± 0.9	1.41 ± 0.15
	7.5% ASt	16.5 ± 1.0	0.75 ± 0.19

Blank disc zone of inhibition < 6 mm.
